# The Association between Apparent Temperature and Hospital Admissions for Cardiovascular Disease in Limpopo Province, South Africa

**DOI:** 10.3390/ijerph20010116

**Published:** 2022-12-22

**Authors:** Jacqueline Lisa Bühler, Shreya Shrikhande, Thandi Kapwata, Guéladio Cissé, Yajun Liang, Hugo Pedder, Marek Kwiatkowski, Zamantimande Kunene, Angela Mathee, Nasheeta Peer, Caradee Y. Wright

**Affiliations:** 1 Department of Global Public Health, Karolinska Institutet, 171 77 Stockholm, Sweden; 2 Epidemiology and Public Health Department, Swiss Tropical and Public Health Institute, 4123 Allschwil, Switzerland; 3 Faculty of Science, University of Basel, 4001 Basel, Switzerland; 4 Environment and Health Research Unit, South African Medical Research Council, Johannesburg 2094, South Africa; 5 Environmental Health Department, Faculty of Health Sciences, University of Johannesburg, Johannesburg 2094, South Africa; 6 Population Health Sciences, University of Bristol, Bristol BS8 2PS, UK; 7 Non-Communicable Diseases Research Unit, South African Medical Research Council, Durban 4091, South Africa; 8 Department of Medicine, University of Cape Town, Cape Town 7925, South Africa; 9 Environment and Health Research Unit, South African Medical Research Council, Pretoria 0001, South Africa; 10 Department of Geography, Geoinformatics and Meteorology, University of Pretoria, Pretoria 0001, South Africa

**Keywords:** climate change, cardiovascular diseases, apparent temperature, distributed lag non-linear model, rural setting, South Africa, time-series analysis

## Abstract

Cardiovascular diseases (CVDs) have a high disease burden both globally and in South Africa. They have also been found to be temperature-sensitive globally. The association between temperature and CVD morbidity has previously been demonstrated, but little is known about it in South Africa. It is important to understand how changes in temperature in South Africa will affect CVD morbidity, especially in rural regions, to inform public health interventions and adaptation strategies. This study aimed to determine the short-term effect of apparent temperature (T_app_) on CVD hospital admissions in Mopani District, Limpopo province, South Africa. A total of 3124 CVD hospital admissions records were obtained from two hospitals from 1 June 2009 to 31 December 2016. Daily T_app_ was calculated using nearby weather station measurements. The association was modelled using a distributed lag non-linear model with a negative binomial regression over a 21-day lag period. The fraction of morbidity attributable to non-optimal T_app_, i.e., cold (6–25 °C) and warm (27–32 °C) T_app_ was reported. We found an increase in the proportion of admissions due to CVDs for warm and cold T_app_ cumulatively over 21 days. Increasing CVD admissions due to warm T_app_ appeared immediately and lasted for two to four days, whereas the lag-structure for the cold effect was inconsistent. A proportion of 8.5% (95% Confidence Interval (CI): 3.1%, 13.7%) and 1.1% (95% CI: −1.4%, 3.5%) of the total CVD admissions was attributable to cold and warm temperatures, respectively. Warm and cold T_app_ may increase CVD admissions, suggesting that the healthcare system and community need to be prepared in the context of global temperature changes.

## 1. Introduction

Cardiovascular disease (CVD), the leading cause of death globally, is a major threat to human health and well-being. CVD is a broad term used to encompass a group of multi-factorial diseases affecting the structure and function of the heart and the vasculature system. In 2019, CVDs were estimated to account for 32% and 15% of the global mortality and morbidity, respectively [1]. Non-modifiable (age, sex, and genetics), modifiable behavioural risk factors such as physical inactivity; unhealthy dietary patterns; tobacco; and alcohol consumption as well as external determinants, including stress, noise and air pollution, and socio-economic status are known contributors to CVD incidences [1,2,3]. CVDs are a public health issue, especially in low- and middle-income countries (LMICs), with over 75% of global CVD deaths occurring in these regions, partially due to inequitable healthcare services and insufficient awareness of early intervention programmes [1].

Over the years, a growing body of research has found temperature to be an environmental factor affecting CVD mortality [3,4,5,6,7]. The risk of severe illness or morbidity due to CVDs increases at very high or very low ambient temperatures, although this is context-dependant [8,9]. The thermoregulatory response of the cardiovascular system is thought to play a role in driving the temperature-CVD association. As a response to heat stress, the superficial blood vessels dilate, increasing blood flow to the skin, and which increases cardiac workload. With prolonged heat exposure and inability to lose bodily heat by sweating an increase in core temperature occurs. The inability of the cardiac output to compensate for this leads to a cardiovascular event, usually through heat intolerance. A core body temperature above 38 °C results in heat exhaustion with impaired physical and cognitive functions and higher than 40 °C leads to heat stroke and increased risks of organ damage and mortality [3,10,11]. In contrast, exposure to cold causes an increase in the cardiac workload and a sustained increase in systemic blood pressure resulting in cardiovascular dysregulation, through vasoconstriction that reduces blood flow to the heart [3,12]. Further, altered blood constitution and consequent increase in haemo-concentration and fibrinogen as a response to cold, favours coagulation and increases the risk of thromboses [13]. This response is also driven by the cold-related autonomous nervous system activation, occasionally leading to arrhythmias [14].

There are several studies showing the effects of temperature on mortality and morbidity, although fewer tend to focus on the latter [5,6,15,16,17,18]. Studies have shown an increased short-term effect of temperature on CVD mortality; some have shown a V-, U- or J-shaped relationship, which suggests an increase in the risk for CVD above and below an optimal temperature [19,20,21,22]. Optimal temperature is defined as the temperature with the lowest risk for CVD mortality and temperatures above and below this are referred to as warm and cold temperatures, respectively [21,23]. However, with respect to CVDs, optimal temperature and the extent to which temperatures affect them, varies by climate, region and population [21,23].

Taken in the context of anthropogenic climate change, global mean surface temperatures are largely projected to increase, with an increase in temperature extremes, i.e., days of extreme heat and cold, intensifying in frequency and duration [24]. The rapid pace of this global warming has emerged as a major threat to human health and is a developing public health challenge [4]. Non-optimal temperatures are now among the top 10 leading causes of mortality worldwide, according to a recent Global Burden of Disease study [25]. Given the large burden of CVDs and associations between temperature and CVD, there is reason for concern that the prevalence of CVD and short-term effects, such as hospital admissions, may increase in the future with frequent extremes of temperature [16,17,18,26]. The impacts are likely to have the greatest effect in vulnerable communities and in LMICs [27,28]. Physical factors such as rural settings, low educational attainment, and air pollution have been recognized to increase the susceptibility to temperature-related CVDs [29,30,31,32].

South Africa is facing a double burden in this context; warming over South Africa is happening at twice the global rate and ischaemic heart diseases (IHDs) and related co-morbidities like diabetes are the only diseases that have seen an increased prevalence over the past decade [33,34]. In 2019, CVDs accounted for 16% of all deaths and 7% of all disability-adjusted life years (DALYs) in South Africa [33]. People from low socio-economic groups and those living in remote or rural areas are at increased risk for poorer CVD outcomes, partially due to inadequate access to medical facilities compared to their urban counterparts [1,35,36]. Few studies have considered the relation between temperature and mortality or morbidity in South Africa. Using data for 1997 to 2013, a study found that 3.4% of the nation-wide mortality was due to non-optimal temperature, with 3% attributable to cold and 0.4% attributable to warm temperatures [21]. Two studies considered CVD morbidity using hospital admissions data and the effect modification of temperature on the association between air pollution and CVD admissions for different regions of South Africa and reported contrasting findings [31,37]. As the average temperature projections for South Africa suggesting between 4 to 6 °C increases in temperature by 2100, the risk of hospital admissions for CVD-related illnesses attributable to temperature requires further investigation for improved prevention and preparedness [38,39].

Given the variation in findings across and within countries, the aim of this study was to perform an exploratory analysis investigating the short-term association between apparent temperature (T_app_) and CVD hospital admissions in Mopani District Municipality, Limpopo province, South Africa and the proportion of CVD admissions attributable to non-optimal T_app_.

## 2. Materials and Methods

### 2.1. Study Setting

**Figure 1:**
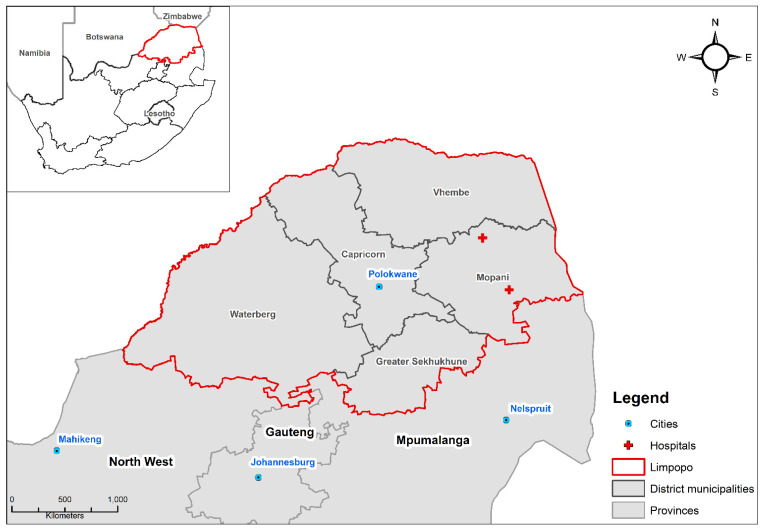
Location of the two hospitals in the study site of Mopani District, Limpopo province, South Africa.

Mopani district is located in Limpopo province in north-eastern South Africa. Mopani district is predominantly rural with high levels of unemployment, with approximately 55% of the population living below the upper poverty line of ZAR 1227 (equivalent to ~73 USD) per month [40]. Climate studies suggest that this region is vulnerable to the health impacts of heat exposure due to projections predicting significant increases in temperature and heatwave events [38,41,42]. Daily hospital admission data was collected from two large hospitals, namely Nkhensani Hospital and Maphutha L. Malatjie (Figure 1) which are two out of six secondary level health-care facilities in the district. The dataset has been used in previous studies to consider diarrhoeal disease [43], respiratory disease [44], malaria and asthma [45] but not CVD hospital admissions.

### 2.2. Data Sources

#### 2.2.1. Health Data

The variables in the hospital admission data included date of admission and discharge, time spent in hospital, age and sex. The dataset was not classified using the International Classification of Disease-10 (ICD-10) codes and only the initial diagnosis on admission was available. Therefore, the ICD-10 IX (diseases on the circulatory system) classification list was employed to identify CVD cases for all ages in consultation with a general medical doctor from South Africa. The list with the broadly classified CVDs used in this study is included in the Supplementary Material (Table S1). While the dataset covered the period of 2002 to 2016, we chose to include only the cases from 2009 to 2016 owing to a high amount of missingness for the period prior to that, partially due to misplaced admission books.

There was a total of 37,090 all-cause admissions from 2009 to 2016. Of that, a total of 4097 CVD admission cases were included in this study, from which 1726 cases were missing the date of admission. As the date of admission was considered to be the outcome date, we imputed the missing information by subtracting the average length of hospital stay (17 days) from the date of discharge (if reported). A total of 43.6% of the cases could be imputed and the remaining 56.4% of the cases with neither date of admission nor date of discharge available were excluded from the analysis (23.6% of the overall sample). From the CVD admissions without missing information on those variables, 51.57% and 48.43% were males and females, and 21.07% and 78.93% were <45 years and ≥45years of age, respectively.

A final sample of 3124 CVD admissions occurring in the study period between 1 June 2009 and 31 December 2016 was included in the analysis. All admission records were aggregated to daily counts and merged with daily T_app_ data (see below). 147 days with no hospital admissions were treated as missing data and excluded from the model.

#### 2.2.2. Weather Data

Meteorological data were obtained from Thohoyandou where a weather station was located approximately 40 km outside of the Mopani district. Data on mean daily temperature (°C) and relative humidity (%) were directly extracted from the dataset. In a previous study, temperatures measured at the Thohoyandou weather station and in dwellings in Giyani (the location of the hospitals) were well correlated (R = 0.98, *p* <0.001), suggesting that meteorological conditions did not vary substantially between the station and the communities under study [44].

Missing meteorological variables were imputed with chained equations followed by predictive mean matching. Variables used to predict missing weather variables were average temperature (T_avrg_), relative humidity (RH), wind speed (WS) and total rainfall. For days where no weather data was available, T_app,_ was calculated using an exponential rolling average of the three days before and after, where more weight was given to T_app_ of more recent days. Out of the 2771 days in the study period, T_app_ was imputed for 19 days with the predictive mean matching method and for two days with the rolling average.

T_app_, a metric that combines temperature and relative humidity, was chosen as the exposure variable, as it ought to represent the ‘real-feel’ of temperature. T_app_ was calculated using the Steadman equation shown below, where hPa is the vapour pressure [46].

$$T_{\mathrm{app}}=T_{\mathrm{avg}}+0.33*\mathrm{hPa}-0.7*\mathrm{WS}-4$$

$$\mathrm{hPa}=\frac{\mathrm{RH}}{100}*6.105*e\frac{\left(17.27*T_{\mathrm{avg}}\right)}{\left(237.7+T_{\mathrm{avg}}\right)}$$

### 2.3. Statistical Analyses

#### 2.3.1. Statistical Model

Following Vicedo-Cabrera et al. [47], we conducted a time-series analysis to quantify the short-term association between T_app_ and the proportion of admissions due to CVDs. The daily share of admissions due to CVDs was modelled with negative binomial regression in the distributed-lag non-linear modelling (DLNM) framework. The negative binomial regression model allowed for overdispersion of daily CVD counts, while DLNM allowed simultaneous modelling of non-linear exposure–response relationships and delayed effects. The model equation is as follows:

$$\mathrm{Log}\;\left({\mathrm{CVD}\;\mathrm{admissions}}_{t}\right)=\;\alpha +\log\left({\mathrm{total}\;\mathrm{admissions}}_{t}\right)+f\left({T_{\mathrm{appt}}}_{t},\beta_{1}\right)+\beta_{2}\mathrm{ns}\left(t,8\mathrm{df}\right)+\eta_{1}{\mathrm{DOW}}_{t}+\epsilon_{t}$$

where 𝛼 is the model intercept, $f\left({T_{\mathrm{appt}}}_{t},\beta_{1}\right)$ is a delayed lag non-linear function incorporating a cross-basis matrix that defines the relationship between T_app_ and log(CVD admissions) at time t, $\beta_{2}\mathrm{ns}\left(t,8\mathrm{df}\right)$ is a vector of coefficients $\beta_{2}$ for a natural spline function of t with 8 degrees of freedom to account for long-term trends, $\eta_{1}$ is a coefficient for ${\mathrm{DOW}}_{t}$ (day of the week at time t and $\epsilon_{t}$ is the residual. $f\left({T_{\mathrm{appt}}}_{t},\beta_{1}\right)$ is modelled by a crossbasis matrix which consists of the exposure–response (T_app_-CVD) and lag-response (lag-CVD) functions.

The exposure–response relationship was modelled with a natural cubic spline with two internal knots at 15 °C and 26 °C. The knot placement was based on how frequently the T_app_ occurred during the study period. The best set of knots in a range of frequent T_app_ were then chosen from among a few candidate specifications using Akaike Information Criterion (AIC) (Table S2).

The lag-response relationship was modelled with a natural cubic spline with three equally spaced internal knots on the log scale, because this allowed for more flexibility at shorter lags in the model where we expected the most pronounced effects. A lag period of 21 days was motivated by previous studies and is considered as the short-term effect of T_app_ on CVD [21,23,48].

The model was adjusted for the log of daily counts of total hospital admissions to reduce the imprecision that could have arisen as a result of the digitization of handwritten admission data. We also adjusted for day of the week (DOW) and time. DOW was modelled as a categorical variable with seven categories while time was modelled as an integer variable using a natural cubic spline with 8 degrees of freedom.

As the interest of this study was to investigate the short-term (21 days) association between T_app_ and CVD admissions, long-term patterns such as hospital access and seasonality, were likely to obscure the short-term association [49]. Hence, by adjusting the model for time, only the short-term variations of CVD admissions caused by T_app_ were investigated.

#### 2.3.2. Relative Risk and Optimal Temperature

The bi-dimensional (exposure-lag-response) relationship was reduced to a one-dimensional (exposure-response) relationship by calculating overall cumulative risk ratios (RR). For each T_app_, the RR cumulated over the whole 21-day lag period was calculated. The RRs were calculated using the optimal T_app_ as the reference. The optimal T_app_ was derived from the overall cumulative exposure-response curve over the 21-day lag period, as suggested by a previous study [50]. It is the lowest point of the overall cumulative curve in a range of frequently occurring T_app_, where the risk for CVD admissions is lowest. T_app_, which occurred infrequently during the study period, were not chosen as the optimal T_app_ even if they had the lowest risk for CVD admissions from the cumulative curve, as cumulative risks at more extreme temperature ranges were imprecisely estimated and likely to be sensitive to the choice of exposure–response function. This means that extreme T_app_ was not chosen as the optimal T_app_, due to their infrequent occurrence during the study period. T_app_ above and below the optimal T_app_ are referred to as warm and cold T_app_, respectively. Non-optimal T_app_ is defined as all cold and warm T_app_s together.

#### 2.3.3. Attributable Fraction

The share of CVD admissions due to non-optimal T_app_ was calculated by summing up the CVD admissions from all days of the series, relative to the optimal T_app_. The ratio with the total CVD admission resulted in the attributable fraction (AF). The AF for cold and hot T_app_ was calculated by summing up the contributions from days colder or hotter than the optimal T_app_, respectively. Empirical 95% confidence intervals (CI) were conducted using the Monte Carlo simulation over 1000 repetitions. All of those calculations were done using the *attrdl.R* script developed by Gasparrini [51].

#### 2.3.4. Sensitivity Analysis

Sensitivity analysis was performed to check the robustness of the model by changing the duration of the lag period and using the datasets without imputed dates of admissions from 1 June 2009 to 31 December 2016 and from 1 January 2002 to 31 December 2016. As studies often use a quasi-Poisson regression model instead of the negative binomial regression model, an additional sensitivity analysis was conducted to compare these two models. In this study, a negative binomial model was chosen over a quasi-Poisson regression model so that AIC is available to discriminate between candidate models, and because it allows for a more flexible relationship between the variance and the mean.

## 3. Results

### Descriptive Results

**Figure 2:**
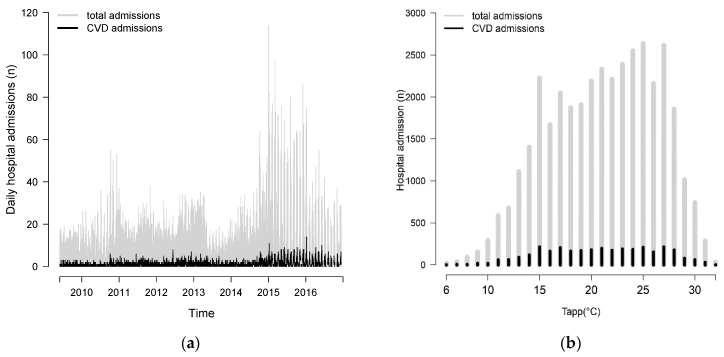
(**a**) The distribution of daily total (N = 37,090) and CVD (N = 3124) hospital admission counts during the study period, and (**b**) the distribution of total and CVD hospital admission counts in relation to T_app_.

**Figure 3:**
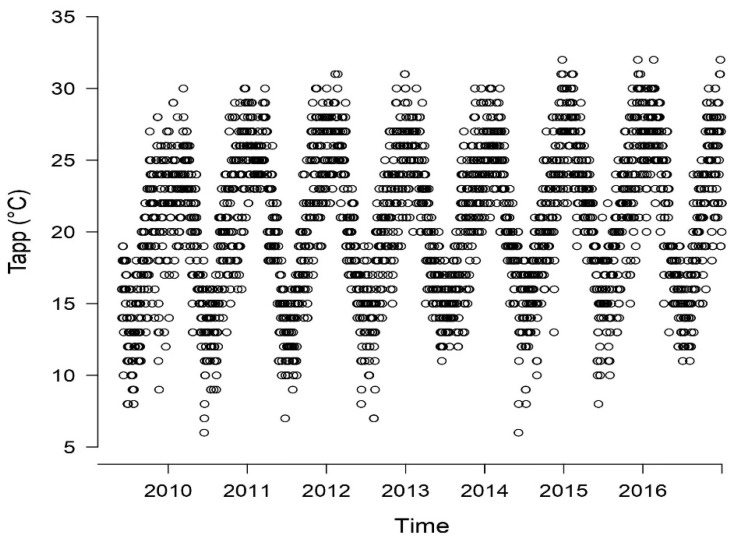
Trends in calculated T_app_ during the study period. Each dot represents the daily T_app_ calculated with weather data from the Thohoyandou weather station.

There were 3124 admissions for CVD during the study period. Figure 2 shows an overview of the distribution pattern of daily CVD hospital admission counts during the study. Figure 2a shows the pattern of daily counts of total and CVD admission during the study period. Seasonal trends were not observed in the daily hospital admissions counts. However, high admission counts were observed between the years 2015 and 2016. Figure 2b shows the distribution of the 37,090 total admissions and 3124 CVD admissions over the T_app_ range. It is visible that, towards the warm T_app_ extremes, the number of total as well as CVD admissions decreased. Seasonal trends of T_app_ ranged from 6 °C to 32 °C, with the 50th percentile at 21 °C (Figure 3). Yearly averages ranged from 19 °C to 21 °C with an overall mean of 20 °C.

**Figure 4:**
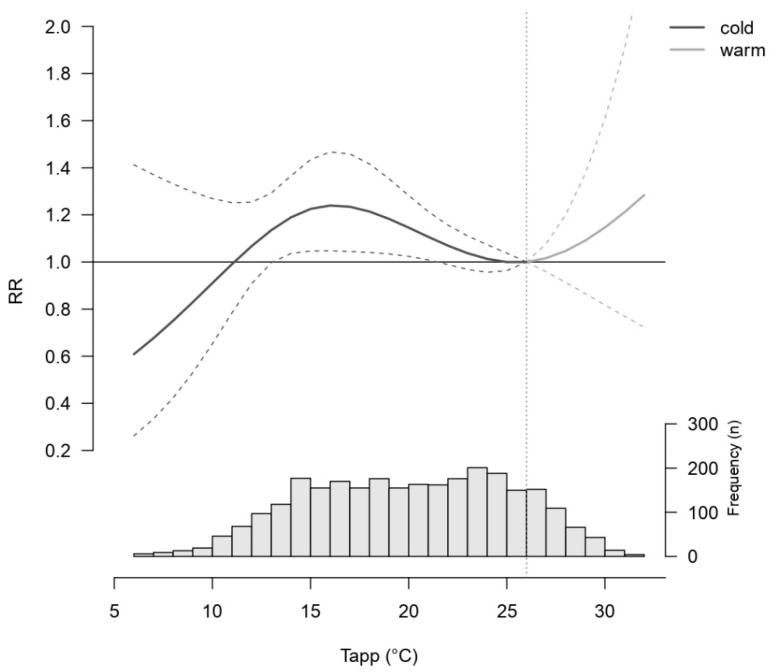
The relative risk (RR) of CVD hospital admissions from apparent temperature (T_app_) accumulated over 21 days of lag. The grey vertical dotted line marks the optimal T_app_ of 26 °C, against which the risk for other T_app_ was compared. The graph represents the RR for cold (dark) and warm (grey) T_app_. The dotted lines represent the 95% confidence interval. The histogram shows the frequency of each T_app_ during the study period.

Figure 4 shows the effect of T_app_ on the risk of CVD admissions cumulated over 21 lag days. The optimal T_app_ is identified at 26 °C (corresponding to the 83rd percentile of T_app_ distribution) and is used as the reference value against which the risk for other T_app_ is compared.

The histogram shows that most days have a T_app_ below the optimal T_app_ of 26 °C. The cumulative curve displays that the risk for CVD admissions increases steadily from 27 °C–32 °C, with 32 °C showing a 33% (95% CI: 0.75, 2.36) increased risk for a CVD admission compared to 26 °C. However, wide 95% CIs reveal the uncertainty of those findings.

Cold T_app_ from 12 °C–24 °C showed an increased risk in CVD admissions relative to 26 °C, with an increase between 14 °C–21 °C (Figure 4). The cold T_app_ with the highest risk for CVD admissions was 16 °C, which showed a 25% (95% CI: 1.05, 1.48) higher risk for CVD admissions cumulated over 21 days compared to 26 °C. On the other hand, cold T_app_ between 6 °C–11 °C seemed to be protective towards CVD admissions accumulated over 21 days, relative to 26 °C. However, the 95% CIs were wide in this T_app_ range due to fewer data points, rendering these particular findings non-significant.

**Figure 5:**
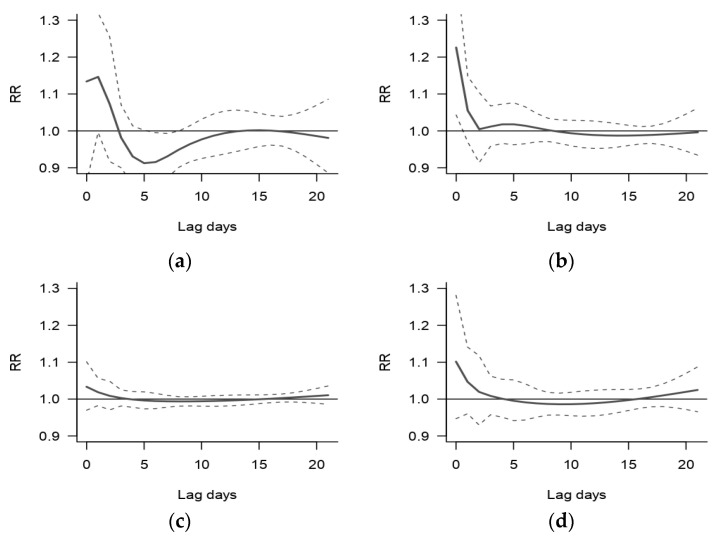
The relative risk (RR) of CVD hospital admissions by lag days at; (**a**) 9 °C; (**b**) 16 °C, (**c**) 28 °C; and (**d**) 30 °C in Limpopo, South Africa. The dotted lines show the 95% CI. The RRs are compared to an optimal T_app_ of 26 °C.

The cumulative graph in Figure 4 also shows that RRs for 25 °C and 26 °C were approximately one, which indicates a ‘comfort zone’, rather than one optimal T_app_ (see extracted RRs and 95% CIs in Table S2). The lag-response structure analysis was conducted for 9 °C, 16 °C, 28 °C and 30 °C relative to the optimal T_app_ of 26 °C (Figure 5).

We observed a reduced risk for CVD admissions at 9 °C after five lag days, however there were few days with this temperature. At 16 °C, 28 °C and 30 °C, there was an immediate peak in the risk for CVD admissions at lag zero. The risk for CVD admissions is the highest for 16 °C, with a significant 23% (95% CI:1.04,1.44) increased risk for CVD admissions compared to 26 °C at lag zero. The increase in risk of 28 °C and 30 °C lasted for two and four days relative to the effect of 26 °C, respectively (see Tables S3 and S4 for the extracted RRs and 95% CIs).

Our sensitivity analyses, presented in the Supplementary Material, changing knot placements, lag durations and regression models showed that our final model was insensitive to these changes. Further detailed summary of the chosen model is presented in Table S5.

Table 1 shows the AF for CVD admissions attributable to non-optimal T_app_ during the study period. 9.5% (95% CI: 3.4%, 15.7%) of the CVD admissions to the two hospitals in Limpopo were attributable to non-optimal T_app_ (Table 1). Cold T_app_ account for most of the total CVD admissions with an 8.5% (95% CI: 3.1%, 13.7%) AF, whereas warm T_app_ account for 1.16% (95% CI: -1.43%, 3.51%) of the total CVD admissions.

## 4. Discussion

We found an association between T_app_ and the share of hospital admissions due to CVDs at both warm and cold non-optimal temperatures. Warm (i.e., 27 °C to 32 °C) as well as cold T_app_ (i.e., 12 °C to 24 °C) had the potential to increase the share of CVD hospital admissions cumulated over 21 days, with cold temperatures being responsible for the biggest fraction of CVD admissions. The effect of warm T_app_ was found to be immediate and short-lived for around two to four days after exposure. Cold T_app_ between 14 °C and 25 °C showed an immediate but long-lasting (5 to 9 days) increased risk while very cold T_app_ between 6 °C and 9 °C showed a delayed (one day) and short-lived (2 days) increased risk for CVD admissions. The seemingly ‘protective’ effect seen at very cold temperatures after the short period of increased risk could potentially be attributable to the harvesting effect, where increased risk of CVD admissions for the most vulnerable population is brought forward in time [52,53]. The initial increased risk of CVD admissions could be attributed to findings that performing physical activity during cold temperatures has been shown to trigger angina pectoris and myocardial infarction and cold temperatures have been found to lead to thrombosis [54]. However, given the wide CI intervals and less days with very cold temperatures, this could also be a statistical artefact. Therefore, more studies are needed from this region to elaborate the findings.

We found a longer lasting cold effect of around 5 to 9 days for T_apps_ between 14 °C and 25 °C was found, which is in accordance with other studies on this topic [55]. Cold temperatures in South Africa have been shown to be responsible for 3.0% of the national mortality proportion and warm temperatures for only 0.4% [21]. Hence, if other diseases are exacerbated by cold temperatures, hospitals might easily be overburdened and can no longer admit patients. This situation could be further complicated by the fact that Limpopo province is one of the provinces with the least established healthcare infrastructure in South Africa [56]. The immediate and short lived effect of warm T_app_ on the risk of CVD hospital admissions found in this study was also observed in other studies conducted in the United States [54], Vietnam [57], Australia [58] and Denmark [59].

In terms of CVD morbidity, our study suggests that the optimal temperature is in the range of 25 °C to 26 °C. The AF from cold T_app_ was found to be responsible for the biggest fraction of the total CVD admissions. This is in accordance with findings of other studies, where cold temperatures were responsible for most of the temperature-related CVD morbidity [60,61,62]. The cold effect might be of greater magnitude compared to the warm effect because housing and lifestyle in a usually warm climate does not adequately protect against cold temperatures. A significant proportion of residents in Mopani district live in poverty and would therefore lack the resources such as electric or gas heaters to protect themselves from cold weather and the associated health impacts on CVDs [56,63,64].

Our study had several strengths. First, we investigated the association between T_app_ and CVD mortality over a long time period and few missing data points, while accounting for environmental confounders such as humidity in the model. Second, the sophisticated modelling approach allowed us to flexibly examine this association by considering both temperature and lag duration at the same time. Third, we were able to demonstrate the burden of both cold and hot non-optimal temperatures, which, to our knowledge, was hitherto not investigated in this region.

This study considered several limitations. Data quality of hospital admission records was inconsistent due to handwritten data reporting systems, leading to potential missing information. To counter this, we modelled the proportion of admissions due to CVDs rather than their absolute number. There is a chance of non-differential outcome misclassification since the CVD data were only classified by a physician’s first clinical assessment upon admission and not medically confirmed. The prevalence of CVDs might be underestimated because the hospital admission records only captured hospitalized CVD cases, thus discounting cases that did not need hospitalization or sought traditional healers. Imputing missing data might have introduced non-differential exposure misclassification. However, sensitivity analysis showed the methods to be robust and the conclusions drawn from the findings to be unaffected. The daily exposure to T_app_ was assumed to be the same for everyone in Limpopo. Potential confounders such as age, sex, socioeconomic status or different types of CVDs, which have been shown to respond differently to temperature, could not be taken into consideration [65]. There may have been some patients who passed away before arriving at the hospital and this would contribute to a possible underestimation. Private hospitals were not considered in this study, potentially excluding a proportion of higher socio-economic status individuals assuming they are more often treated in private hospitals, where access is dependent on affordability [66]. Nevertheless, this concerns a small proportion of the population of the country since the private sector serves only 16% of the entire population of South Africa [66].

To strengthen the findings of this study on the associations between T_app_ and CVD morbidity in Limpopo, further research should gather additional variables to identify the most vulnerable groups, based on factors such as age, sex, type of CVD and socio-economic status. Such studies are important for surveillance systems of population health and for health-care planning. Factors such as gender differences play a major role in the association between temperature and CVDs, especially in conjunction with age. Men and women have different physiological and thermoregulatory responses following heat and cold exposure, with men generally being able to tolerate heat better and females being more vulnerable to heat [67,68,69,70]. The information would better inform interventions, e.g., to develop early warning systems of the effects of cold and heat to prepare health professionals and the communities to encourage them to take precautions based on their predisposed vulnerabilities. There is a need to improve surveillance systems to track diseases like CVD and especially those likely to be influenced by climate change. Hospital admissions data collection systems should be digitized, and physicians and hospital staff should be trained to record patient details and diagnosis in a uniform manner. Such systems will not only benefit tracking within the healthcare systems but will also be invaluable for research purposes to generate evidence for decision and policy making.

## 5. Conclusions

In this study, the non-linear association between T_app_ and the relative frequency of CVD admissions in Limpopo province of South Africa was characterised. We demonstrated that both cold and hot non-optimal T_app_ is associated with an increase in the risk of CVD morbidity, with cold non-optimal T_app_ attributable to a larger fraction of cases. The warm effect was immediate and short lived while the cold effect had a delayed presentation and lasted longer. Our study provides a more nuanced glimpse into the temperature attributable CVD issues facing Limpopo in the coming decades in light of climate change.

## Figures and Tables

**Table 1:** The attributable fraction of CVD hospital admissions (N = 3124) from the two hospitals during the study period due to non-optimal cold and warm T_app_. The lower and upper 95% confidence intervals (CI) are presented.

	AF	Lower 95% CI	Upper 95% CI
Non-optimal Tapps	9.54%	3.47%	15.73%
Cold Tapps	8.5%	3.16%	13.72%
Warm Tapps	1.16%	−1.43%	3.51%

## Data Availability

Weather data can be obtained from the South African Weather Service and the codes used in this study are available upon request from the authors.

## Supplementary Materials

The following supporting information can be downloaded at: https://www.mdpi.com/article/10.3390/ijerph20010116/s1, Table S1: Definitive list of cardiovascular diseases considered in this study from two public hospitals in Limpopo, South Africa; Table S2: Knot placement at different apparent temperatures (Tapp) and the corresponding Akaike Information Criteria (AIC) for the negative binomial regression model and distributed lag non-linear model; Table S3: The relative risk (RR) of apparent temperature (Tapp) on cardiovascular disease hospital admissions cumulated over 21 days of lag, relative to 26 °C in Limpopo, South Africa (N = 3124). The upper (u95) and lower (l95) bound of the 95% confidence intervals are presented. The frequency (Freq.) of each Tapp occurring throughout the study period from 1 June 2009 until 31 December 2016 in Limpopo is shown; Table S4: The relative risk (RR) of cardiovascular disease hospital admissions in Limpopo, South Africa, by lag days at specific apparent temperatures (N = 3124). The tables show the upper (u95) and lower (l95) bounds of the 95% confidence interval. The RRs are relative to 26 °C; Table S5: Model coefficients, standard errors (SE) and *p*-values for the negative binomial regression model (N = 3124). v1, v2 and v3 correspond to the gradient of the exposure- response association in the first, second and third interval of a spline, respectively. “l1” corresponds to the gradient of the lagged association in the first interval of a spline, “l2” to that in the second interval, and so on. The coefficients for the adjustment of days of the week, long term trends and total admissions are also represented; Figure S1: A descriptive overview of the distribution of daily total (N = 57,619) and cardiovascular disease (CVD) (N = 4368) hospital admission counts from 1 January 2002 until 31 December 2016 from two public hospitals in Limpopo, South Africa; Figure S2: The relative risk (RR) of cardiovascular disease hospital admissions by apparent temperature (Tapp) cumulated over 21 days of lag, relative to 26 °C (N = 3124) in Limpopo, South Africa. The black and grey thick lines represent the RR using a negative binomial regression model and a quasi-poisson regression model, respectively. The dotted lines represent the 95% confidence intervals and the grey vertical dotted line marks the optimum Tapp at 26 °C. The histogram at the bottom shows the frequency of each Tapp occurring in Limpopo between 1 June 2009 and 31 December 2016; Figure S3: The relative risk (RR) of cardiovascular disease (CVD) hospital admissions by apparent temperature (Tapp) cumulated over different lag periods, relative to 26 °C in Limpopo, South Africa (N = 3124). The black and grey thick lines show the effect of apparent temperature (Tapp) on CVD hospital admissions cumulated over 21 and 10 days of lag, respectively. The dotted lines represent the 95% confidence intervals and the grey vertical dotted line marks the optimum Tapp at 26 °C. The histogram at the bottom shows the frequency of each Tapp occurring in Limpopo between 1 June 2009 and 31 December 2016; Figure S4: The relative risk (RR) of cardiovascular disease hospital admissions by apparent temperature (Tapp) cumulated over 21 days of lag, relative to 26 °C in Limpopo, South Africa. The black and grey thick lines represent the RR using the imputed dataset (N = 3124) and raw dataset (N = 2371), respectively. The dotted lines represent the 95% confidence intervals and the grey vertical dotted line marks the optimum Tapp at 26 °C. The histogram at the bottom shows the frequency of each Tapp occurring in Limpopo between 1 June 2009 and 31 December 2016; Figure S5: The relative risk (RR) of cardiovascular disease hospital admissions by apparent temperature (Tapp) cumulated over 21 days of lag, relative to 26 °C in Limpopo, South Africa. The black and grey thick lines represent the RR using data from 1 June 2009 until 31 December 2016 (N = 3124) and non-imputed data from 1.January 2002 until 31.December 2016 (N = 2642), respectively. The dotted lines represent the 95% confidence intervals and the grey vertical dotted line marks the optimum Tapp at 26 °C. The histogram at the bottom shows the frequency of each Tapp occurring in Limpopo between 1 January 2002 and 31 December 2016.
