# Supplementary material for: The Association between Apparent Temperature and Hospital Admissions for Cardiovascular Disease in Limpopo Province, South Africa
Source: Int J Environ Res Public Health. 2022 Dec 22;20(1):116. doi: 10.3390/ijerph20010116 (PMC9820030; doi:10.3390/ijerph20010116)
Supplement: Supplementary file 1 [file ijerph-20-00116-s001.zip › ijerph-2040782-supplementary.pdf]

Supplementary Material

Table S1: Definitive list of cardiovascular diseases considered in this study from two public hospitals in Limpopo, South Africa.

- Coronary artery disease
- Angina
- Myocardial infarction
- Stroke
- Heart failure
- Hypertensive heart failure
- Rhematic heart failure
- Cardiomyopathy
- Heart murmurs
- Congenital heart failure
- Valvular heart disease
- Carditis
- aortic aneurism
- peripheral artery disease
- thromboembolic dieses
- congestive cardiac failure (CCF)
- cerebrovascular accident (CVA)
- Atherosclerosis
- High blood pressure
- Hypertension
- High cholesterol/cholesterol
- Deep Vein Thrombosis/Venous thrombosis/ DVT

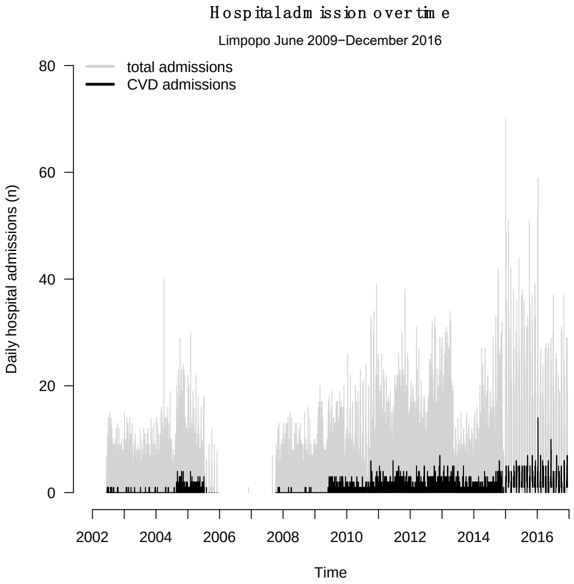

Figure S1: A descriptive overview of the distribution of daily total (N=57,619) and cardiovascular disease (CVD) (N=4,368) hospital admission counts from 1.January 2002 until 31. December 2016 from two public hospitals in Limpopo, South Africa.

Table S2: Knot placement at different apparent temperatures (Tapp) and the corresponding Akaike Information Criteria (AIC) for the negative binomial regression model and distributed lag non-linear model.

**Tapp**	**AIC**
15°C, 26°C	6684.9
17°C, 26°C	6685.1
18°C, 26°C	6685.4
15°C, 20°C, 25°C	6693.3

Table S3: The relative risk (RR) of apparent temperature (Tapp) on cardiovascular disease hospital admissions cumulated over 21 days of lag, relative to 26°C in Limpopo, South Africa (N=3,124). The upper (u95) and lower (l95) bound of the 95% confidence intervals are presented. The frequency (Freq.) of each Tapp occurring throughout the study period from 1.June 2009 until 31.December 2016 in Limpopo is shown.

Tapp	RR	l95	u95	Freq.
6°C	0.59	0.25	1.37	2
7°C	0.66	0.32	1.33	4
8°C	0.73	0.41	1.30	9
9°C	0.82	0.52	1.28	13
10°C	0.90	0.65	1.26	19
11°C	0.99	0.78	1.25	46
12°C	1.07	0.91	1.25	68
13°C	1.14	1.00	1.3	97
14°C	1.20	1.04	1.38	118
15°C	1.24	1.05	1.45	177
16°C	1.25	1.05	1.48	151
17°C	1.24	1.05	1.47	169
18°C	1.22	1.05	1.43	154
19°C	1.19	1.04	1.36	176
20°C	1.15	1.03	1.29	156
21°C	1.11	1.01	1.22	163
22°C	1.07	0.99	1.16	163
23°C	1.04	0.97	1.11	177
24°C	1.01	0.95	1.07	199
25°C	1.00	0.96	1.04	188
26°C	1.00	1.00	1.00	155
27°C	1.02	0.96	1.08	153
28°C	1.06	0.92	1.21	109
29°C	1.11	0.88	1.39	66
30°C	1.17	0.83	1.64	42
31°C	1.24	0.79	1.96	14
32°C	1.33	0.75	2.36	4

Table S4: The relative risk (RR) of cardiovascular disease hospital admissions in Limpopo, South Africa, by lag days at specific apparent temperatures (N=3,124). The tables show the upper (u95) and lower (l95) bounds of the 95% confidence interval. The RRs are relative to 26°C.

	6°*C*			7°*C*			8°*C*			9°*C*			10°*C*			11°*C*			12°*C*			13°*C*		
**Lag days**	**RR**	**l95**	**u95**	**RR**	**l95**	**u95**	**RR**	**l95**	**u95**	**RR**	**l95**	**u95**	**RR**	**l95**	**u95**	**RR**	**l95**	**u95**	**RR**	**l95**	**u95**	**RR**	**l95**	**u95**
**0**	1.01	0.67	1.53	1.05	0.73	1.51	1.09	0.80	1.49	1.13	0.86	1.48	1.16	0.91	1.48	1.19	0.96	1.48	1.22	1.00	1.48	1.23	1.02	1.49
**1**	1.18	0.96	1.46	1.17	0.97	1.40	1.15	0.98	1.35	1.14	0.99	1.31	1.12	0.99	1.27	1.11	0.99	1.24	1.10	0.99	1.21	1.08	0.98	1.19
**2**	1.11	0.88	1.40	1.10	0.90	1.35	1.08	0.91	1.29	1.07	0.91	1.25	1.05	0.92	1.21	1.04	0.92	1.18	1.03	0.92	1.16	1.02	0.92	1.14
**3**	0.96	0.84	1.09	0.97	0.86	1.08	0.98	0.88	1.08	0.98	0.90	1.07	0.99	0.92	1.07	1.00	0.93	1.07	1.00	0.94	1.07	1.01	0.95	1.07
**4**	0.88	0.77	1.00	0.90	0.80	1.00	0.92	0.83	1.01	0.93	0.86	1.02	0.95	0.88	1.02	0.97	0.91	1.03	0.98	0.93	1.05	1.00	0.94	1.06
**5**	0.85	0.74	0.98	0.87	0.77	0.99	0.89	0.80	1.00	0.92	0.84	1.01	0.94	0.87	1.02	0.96	0.89	1.03	0.98	0.92	1.04	0.99	0.93	1.06
**6**	0.86	0.76	0.97	0.88	0.79	0.98	0.90	0.82	0.99	0.92	0.85	1.00	0.94	0.87	1.01	0.96	0.90	1.02	0.98	0.92	1.03	0.99	0.94	1.05
**7**	0.89	0.80	0.98	0.90	0.83	0.98	0.92	0.85	0.99	0.93	0.88	1.00	0.95	0.90	1.00	0.96	0.92	1.01	0.98	0.93	1.02	0.99	0.95	1.03
**8**	0.92	0.85	0.99	0.93	0.87	1.00	0.94	0.88	1.00	0.95	0.90	1.00	0.96	0.92	1.00	0.97	0.93	1.01	0.98	0.95	1.02	0.99	0.95	1.02
**9**	0.95	0.88	1.02	0.95	0.89	1.02	0.96	0.90	1.02	0.96	0.92	1.01	0.97	0.93	1.01	0.98	0.94	1.02	0.98	0.95	1.02	0.99	0.95	1.02
**10**	0.97	0.89	1.05	0.97	0.90	1.04	0.97	0.92	1.04	0.98	0.93	1.03	0.98	0.93	1.03	0.98	0.94	1.02	0.98	0.95	1.02	0.99	0.95	1.03
**11**	0.99	0.90	1.07	0.99	0.91	1.06	0.99	0.92	1.05	0.99	0.93	1.04	0.99	0.94	1.04	0.99	0.94	1.03	0.99	0.95	1.03	0.99	0.95	1.03
**12**	1.00	0.92	1.09	1.00	0.92	1.08	0.99	0.93	1.06	0.99	0.94	1.05	0.99	0.94	1.04	0.99	0.94	1.04	0.99	0.95	1.03	0.99	0.95	1.03
**13**	1.01	0.93	1.10	1.00	0.93	1.08	1.00	0.94	1.07	1.00	0.94	1.05	0.99	0.95	1.04	0.99	0.95	1.04	0.99	0.95	1.03	0.99	0.95	1.03
**14**	1.01	0.94	1.09	1.01	0.94	1.08	1.00	0.95	1.06	1.00	0.95	1.05	1.00	0.95	1.04	0.99	0.95	1.03	0.99	0.95	1.03	0.99	0.95	1.02
**15**	1.01	0.94	1.09	1.01	0.95	1.07	1.00	0.95	1.06	1.00	0.96	1.05	1.00	0.96	1.04	0.99	0.96	1.03	0.99	0.96	1.02	0.99	0.96	1.02
**16**	1.01	0.95	1.08	1.01	0.95	1.06	1.00	0.96	1.05	1.00	0.96	1.04	1.00	0.96	1.03	0.99	0.96	1.02	0.99	0.96	1.02	0.99	0.96	1.01
**17**	1.00	0.94	1.07	1.00	0.95	1.06	1.00	0.95	1.05	1.00	0.96	1.04	0.99	0.96	1.03	0.99	0.96	1.02	0.99	0.97	1.02	0.99	0.97	1.01
**18**	1.00	0.92	1.08	1.00	0.93	1.06	0.99	0.94	1.05	0.99	0.95	1.04	0.99	0.95	1.03	0.99	0.96	1.03	0.99	0.96	1.02	0.99	0.96	1.02
**19**	0.99	0.90	1.09	0.99	0.91	1.08	0.99	0.92	1.06	0.99	0.93	1.05	0.99	0.94	1.05	0.99	0.94	1.04	0.99	0.95	1.04	0.99	0.95	1.03
**20**	0.98	0.87	1.11	0.98	0.88	1.09	0.98	0.90	1.08	0.99	0.91	1.07	0.99	0.92	1.06	0.99	0.93	1.05	0.99	0.94	1.05	0.99	0.94	1.05
**21**	0.97	0.83	1.13	0.97	0.85	1.11	0.98	0.87	1.10	0.98	0.89	1.09	0.99	0.90	1.08	0.99	0.91	1.07	0.99	0.92	1.07	0.99	0.93	1.07

	**14°*C***			**15°*C***			**16°*C***			**17°*C***			**18°*C***			**19°*C***			**20°*C***			**21°*C***		
**Lag days**	**RR**	**l95**	**u95**	**RR**	**l95**	**u95**	**RR**	**l95**	**u95**	**RR**	**l95**	**u95**	**RR**	**l95**	**u95**	**RR**	**l95**	**u95**	**RR**	**l95**	**u95**	**RR**	**l95**	**u95**
**0**	1.24	1.04	1.48	1.24	1.05	1.47	1.23	1.04	1.44	1.20	1.04	1.40	1.18	1.03	1.34	1.14	1.02	1.29	1.11	1.01	1.23	1.08	0.99	1.17
**1**	1.07	0.98	1.17	1.06	0.97	1.15	1.04	0.96	1.13	1.03	0.96	1.12	1.02	0.95	1.10	1.01	0.95	1.08	1.01	0.95	1.06	1.00	0.96	1.04
**2**	1.01	0.91	1.12	1.00	0.91	1.11	1.00	0.91	1.09	0.99	0.91	1.08	0.99	0.92	1.07	0.99	0.92	1.06	0.99	0.93	1.05	0.99	0.94	1.04
**3**	1.01	0.95	1.07	1.01	0.96	1.07	1.01	0.96	1.07	1.01	0.96	1.06	1.01	0.97	1.06	1.01	0.97	1.05	1.01	0.98	1.04	1.01	0.98	1.03
**4**	1.01	0.96	1.07	1.02	0.97	1.07	1.02	0.97	1.08	1.03	0.98	1.08	1.03	0.98	1.07	1.03	0.99	1.07	1.02	0.99	1.06	1.02	0.99	1.05
**5**	1.01	0.95	1.07	1.02	0.96	1.08	1.03	0.97	1.08	1.03	0.98	1.08	1.03	0.99	1.08	1.03	0.99	1.07	1.03	0.99	1.07	1.02	0.99	1.05
**6**	1.00	0.95	1.06	1.01	0.96	1.07	1.02	0.97	1.07	1.03	0.98	1.07	1.03	0.99	1.07	1.03	0.99	1.07	1.03	0.99	1.06	1.02	1.00	1.05
**7**	1.00	0.96	1.04	1.01	0.97	1.05	1.01	0.98	1.05	1.02	0.98	1.05	1.02	0.99	1.05	1.02	0.99	1.05	1.02	1.00	1.04	1.02	1.00	1.04
**8**	1.00	0.96	1.03	1.00	0.97	1.03	1.01	0.98	1.04	1.01	0.98	1.04	1.01	0.99	1.04	1.01	0.99	1.04	1.01	0.99	1.03	1.01	1.00	1.03
**9**	0.99	0.96	1.03	1.00	0.96	1.03	1.00	0.97	1.03	1.00	0.97	1.03	1.00	0.98	1.03	1.01	0.98	1.03	1.01	0.99	1.03	1.01	0.99	1.02
**10**	0.99	0.95	1.03	0.99	0.96	1.03	0.99	0.96	1.03	1.00	0.97	1.03	1.00	0.97	1.03	1.00	0.98	1.03	1.00	0.98	1.02	1.00	0.99	1.02
**11**	0.99	0.95	1.03	0.99	0.95	1.03	0.99	0.95	1.03	0.99	0.96	1.03	0.99	0.96	1.03	1.00	0.97	1.02	1.00	0.98	1.02	1.00	0.98	1.02
**12**	0.99	0.95	1.03	0.99	0.95	1.03	0.99	0.95	1.02	0.99	0.96	1.02	0.99	0.96	1.02	0.99	0.97	1.02	1.00	0.97	1.02	1.00	0.98	1.02
**13**	0.99	0.95	1.02	0.99	0.95	1.02	0.99	0.95	1.02	0.99	0.96	1.02	0.99	0.96	1.02	0.99	0.97	1.02	0.99	0.97	1.02	1.00	0.98	1.01
**14**	0.99	0.95	1.02	0.99	0.95	1.02	0.99	0.95	1.02	0.99	0.96	1.02	0.99	0.96	1.01	0.99	0.97	1.01	0.99	0.97	1.01	0.99	0.98	1.01
**15**	0.99	0.96	1.02	0.99	0.96	1.01	0.99	0.96	1.01	0.99	0.96	1.01	0.99	0.96	1.01	0.99	0.97	1.01	0.99	0.97	1.01	0.99	0.98	1.01
**16**	0.99	0.96	1.01	0.99	0.96	1.01	0.99	0.96	1.01	0.99	0.97	1.01	0.99	0.97	1.01	0.99	0.97	1.01	0.99	0.98	1.01	0.99	0.98	1.00
**17**	0.99	0.96	1.01	0.99	0.96	1.01	0.99	0.96	1.01	0.99	0.97	1.01	0.99	0.97	1.01	0.99	0.97	1.01	0.99	0.98	1.01	0.99	0.98	1.00
**18**	0.99	0.96	1.02	0.99	0.96	1.02	0.99	0.96	1.02	0.99	0.96	1.02	0.99	0.97	1.01	0.99	0.97	1.01	0.99	0.97	1.01	0.99	0.98	1.01
**19**	0.99	0.95	1.03	0.99	0.95	1.03	0.99	0.95	1.03	0.99	0.96	1.03	0.99	0.96	1.02	0.99	0.96	1.02	0.99	0.97	1.02	0.99	0.97	1.01
**20**	0.99	0.94	1.05	0.99	0.94	1.05	0.99	0.95	1.05	0.99	0.95	1.04	0.99	0.95	1.04	0.99	0.96	1.03	0.99	0.96	1.02	0.99	0.97	1.02
**21**	1.00	0.93	1.07	1.00	0.93	1.07	1.00	0.94	1.06	1.00	0.94	1.06	1.00	0.94	1.05	0.99	0.95	1.04	0.99	0.96	1.03	0.99	0.96	1.02

	23°*C*			24°*C*			25°*C*			26°*C*			27°*C*			28°*C*			29°*C*			30°*C*		
**Lag days**	**RR**	**l95**	**u95**	**RR**	**l95**	**u95**	**RR**	**l95**	**u95**	**RR**	**l95**	**u95**	**RR**	**l95**	**u95**	**RR**	**l95**	**u95**	**RR**	**l95**	**u95**	**RR**	**l95**	**u95**
**0**	1.03	0.97	1.08	1.01	0.97	1.05	1.11	1.01	1.23	1.00	1.00	1.00	1.01	0.98	1.04	1.03	0.97	1.10	1.06	0.96	1.18	1.10	0.95	1.28
**1**	0.99	0.96	1.02	0.99	0.97	1.01	1.01	0.95	1.06	1.00	1.00	1.00	1.01	0.99	1.02	1.02	0.98	1.06	1.03	0.97	1.10	1.05	0.96	1.14
**2**	0.99	0.96	1.02	0.99	0.97	1.02	0.99	0.93	1.05	1.00	1.00	1.00	1.00	0.99	1.02	1.01	0.97	1.05	1.01	0.95	1.08	1.02	0.93	1.12
**3**	1.00	0.98	1.02	1.00	0.99	1.01	1.01	0.98	1.04	1.00	1.00	1.00	1.00	0.99	1.01	1.00	0.98	1.03	1.01	0.97	1.04	1.01	0.96	1.06
**4**	1.01	0.99	1.03	1.01	0.99	1.02	1.02	0.99	1.06	1.00	1.00	1.00	1.00	0.99	1.01	1.00	0.98	1.02	1.00	0.97	1.04	1.00	0.95	1.06
**5**	1.01	0.99	1.03	1.01	0.99	1.02	1.03	0.99	1.07	1.00	1.00	1.00	1.00	0.99	1.01	1.00	0.97	1.02	1.00	0.96	1.04	1.00	0.94	1.05
**6**	1.01	1.00	1.03	1.01	1.00	1.02	1.03	0.99	1.06	1.00	1.00	1.00	1.00	0.99	1.01	0.99	0.97	1.02	0.99	0.96	1.03	0.99	0.94	1.04
**7**	1.01	1.00	1.02	1.01	1.00	1.02	1.02	1.00	1.04	1.00	1.00	1.00	1.00	0.99	1.00	0.99	0.98	1.01	0.99	0.97	1.02	0.99	0.95	1.03
**8**	1.01	1.00	1.02	1.01	1.00	1.01	1.01	0.99	1.03	1.00	1.00	1.00	1.00	0.99	1.00	0.99	0.98	1.01	0.99	0.97	1.01	0.99	0.96	1.02
**9**	1.01	1.00	1.02	1.00	1.00	1.01	1.01	0.99	1.03	1.00	1.00	1.00	1.00	0.99	1.00	0.99	0.98	1.01	0.99	0.97	1.01	0.99	0.96	1.02
**10**	1.00	0.99	1.01	1.00	1.00	1.01	1.00	0.98	1.02	1.00	1.00	1.00	1.00	0.99	1.00	0.99	0.98	1.01	0.99	0.97	1.01	0.99	0.95	1.02
**11**	1.00	0.99	1.01	1.00	0.99	1.01	1.00	0.98	1.02	1.00	1.00	1.00	1.00	0.99	1.00	0.99	0.98	1.01	0.99	0.97	1.01	0.99	0.95	1.02
**12**	1.00	0.99	1.01	1.00	0.99	1.01	1.00	0.97	1.02	1.00	1.00	1.00	1.00	0.99	1.00	1.00	0.98	1.01	0.99	0.97	1.02	0.99	0.95	1.02
**13**	1.00	0.99	1.01	1.00	0.99	1.01	0.99	0.97	1.02	1.00	1.00	1.00	1.00	0.99	1.01	1.00	0.98	1.01	0.99	0.97	1.02	0.99	0.96	1.03
**14**	1.00	0.99	1.01	1.00	0.99	1.01	0.99	0.97	1.01	1.00	1.00	1.00	1.00	0.99	1.01	1.00	0.99	1.01	1.00	0.97	1.02	0.99	0.96	1.03
**15**	1.00	0.99	1.01	1.00	0.99	1.00	0.99	0.97	1.01	1.00	1.00	1.00	1.00	0.99	1.01	1.00	0.99	1.01	1.00	0.98	1.02	1.00	0.97	1.03
**16**	1.00	0.99	1.00	1.00	0.99	1.00	0.99	0.98	1.01	1.00	1.00	1.00	1.00	1.00	1.01	1.00	0.99	1.01	1.00	0.98	1.02	1.00	0.98	1.03
**17**	0.99	0.99	1.00	1.00	0.99	1.00	0.99	0.98	1.01	1.00	1.00	1.00	1.00	1.00	1.01	1.00	0.99	1.01	1.01	0.99	1.02	1.01	0.98	1.03
**18**	0.99	0.98	1.00	1.00	0.99	1.00	0.99	0.97	1.01	1.00	1.00	1.00	1.00	1.00	1.01	1.01	0.99	1.02	1.01	0.99	1.03	1.01	0.98	1.04
**19**	0.99	0.98	1.01	1.00	0.99	1.00	0.99	0.97	1.02	1.00	1.00	1.00	1.00	1.00	1.01	1.01	0.99	1.02	1.01	0.99	1.04	1.02	0.98	1.06
**20**	0.99	0.98	1.01	0.99	0.98	1.01	0.99	0.96	1.02	1.00	1.00	1.00	1.00	1.00	1.01	1.01	0.99	1.03	1.02	0.98	1.05	1.02	0.98	1.07
**21**	0.99	0.97	1.01	0.99	0.98	1.01	0.99	0.96	1.03	1.00	1.00	1.00	1.01	0.99	1.02	1.01	0.99	1.04	1.02	0.98	1.06	1.03	0.97	1.09

Lag days	31°*C* RR	l95	u95	32°*C* RR	l95	u95
**0**	1.15	0.94	1.40	1.19	0.93	1.54
**1**	1.06	0.95	1.19	1.08	0.93	1.25
**2**	1.03	0.91	1.16	1.03	0.88	1.20
**3**	1.01	0.95	1.09	1.02	0.93	1.11
**4**	1.00	0.94	1.08	1.01	0.93	1.10
**5**	1.00	0.93	1.07	1.00	0.91	1.10
**6**	0.99	0.93	1.06	0.99	0.91	1.08
**7**	0.99	0.94	1.04	0.98	0.92	1.05
**8**	0.98	0.94	1.02	0.98	0.93	1.03
**9**	0.98	0.94	1.02	0.98	0.93	1.03
**10**	0.98	0.94	1.02	0.98	0.93	1.03
**11**	0.98	0.94	1.03	0.98	0.92	1.04
**12**	0.98	0.94	1.03	0.98	0.92	1.04
**13**	0.99	0.94	1.03	0.98	0.93	1.04
**14**	0.99	0.95	1.03	0.99	0.94	1.04
**15**	1.00	0.96	1.04	0.99	0.95	1.04
**16**	1.00	0.97	1.04	1.00	0.96	1.05
**17**	1.01	0.97	1.04	1.01	0.97	1.06
**18**	1.02	0.98	1.06	1.02	0.97	1.07
**19**	1.02	0.97	1.08	1.03	0.96	1.10
**20**	1.03	0.97	1.10	1.04	0.96	1.12
**21**	1.04	0.96	1.12	1.05	0.95	1.16

Table S5: Model coefficients, standard errors (SE) and p-values for the negative binomial regression model (N=3,124). v1, v2 and v3 correspond to the gradient of the exposure-response association in the first, second and third interval of a spline, respectively. "l1" corresponds to the gradient of the lagged association in the first interval of a spline, "l2" to that in the second interval, and so on. The coefficients for the adjustment of days of the week, long term trends and total admissions are also represented.

Parameter	Coefficient	SE	p-value
Intercept	-3.15	0.473	<0.001
Mean Temp v1.l1	-0.158	0.183	0.389
Mean Temp v1.l2	0.156	0.105	0.138
Mean Temp v1.l3	0.11	0.115	0.341
Mean Temp v1.l4	-0.275	0.147	0.061
Mean Temp v1.l5	0.154	0.106	0.145
Mean Temp v2.l1	-0.613	0.488	0.209
Mean Temp v2.l2	0.622	0.3	0.038
Mean Temp v2.l3	-0.343	0.31	0.269
Mean Temp v2.l4	0.204	0.416	0.624
Mean Temp v2.l5	-0.049	0.295	0.868
Mean Temp v3.l1	0.007	0.171	0.968
Mean Temp v3.l2	0.023	0.1	0.815
Mean Temp v3.l3	-0.013	0.105	0.904
Mean Temp v3.l4	-0.037	0.136	0.785
Mean Temp v3.l5	0.082	0.096	0.391
Monday	0.069	0.068	0.311
Saturday	0.057	0.071	0.416
Sunday	0.071	0.073	0.33
Thursday	0.019	0.069	0.783
Tuesday	0.101	0.072	0.161
Wednesday	0.139	0.071	0.05
LongTerm v1	0.405	0.17	0.017
LongTerm v2	0.034	0.22	0.878
LongTerm v3	0.806	0.189	<0.001
LongTerm v4	-0.247	0.211	0.241
LongTerm v5	0.552	0.194	0.004
LongTerm v6	0.895	0.143	<0.001
LongTerm v7	0.734	0.391	0.061
LongTerm v8	0.867	0.141	<0.001
Total admissions	0.855	0.03	<0.001

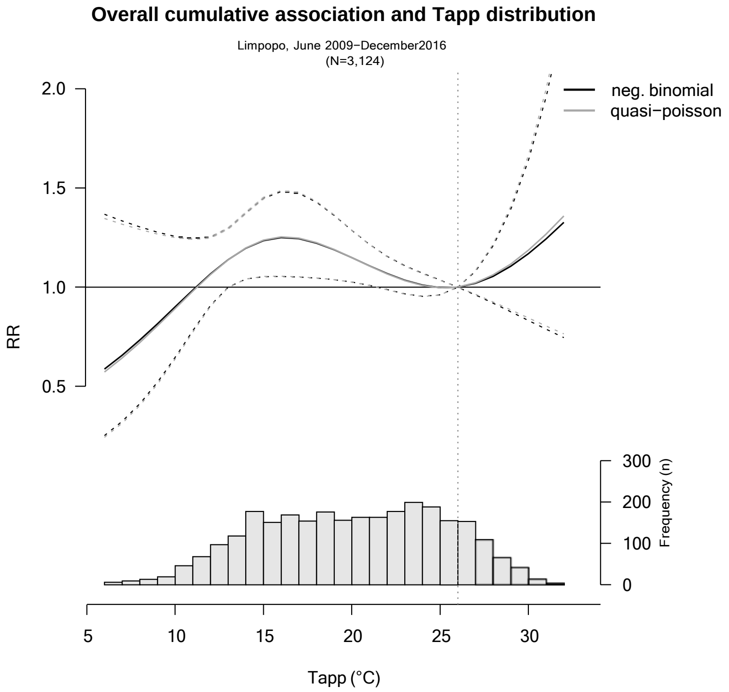

Figure S2: The relative risk (RR) of cardiovascular disease hospital admissions by apparent temperature (Tapp) cumulated over 21 days of lag, relative to 26°C (N=3,124) in Limpopo, South Africa. The black and grey thick lines represent the RR using a negative binomial regression model and a quasi-poisson regression model, respectively. The dotted lines represent the 95% confidence intervals and the grey vertical dotted line marks the optimum Tapp at 26°C. The histogram at the bottom shows the frequency of each Tapp occurring in Limpopo between 1.June 2009 and 31.December 2016.

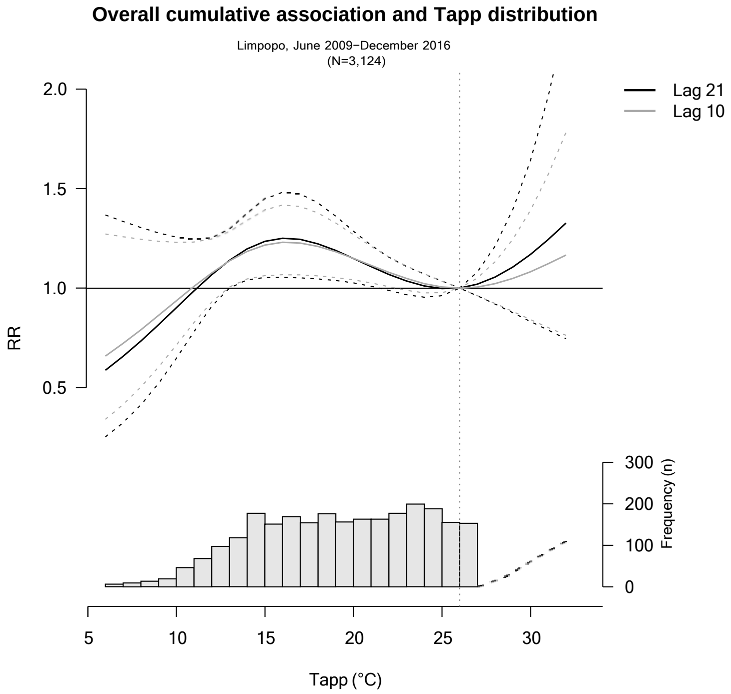

Figure S3: The relative risk (RR) of cardiovascular disease (CVD) hospital admissions by apparent temperature (Tapp) cumulated over different lag periods, relative to 26°C in Limpopo, South Africa (N=3,124). The black and grey thick lines show the effect of apparent temperature (Tapp) on CVD hospital admissions cumulated over 21 and 10 days of lag, respectively. The dotted lines represent the 95% confidence intervals and the grey vertical dotted line marks the optimum Tapp at 26°C. The histogram at the bottom shows the frequency of each Tapp occurring in Limpopo between 1.June 2009 and 31.December 2016.

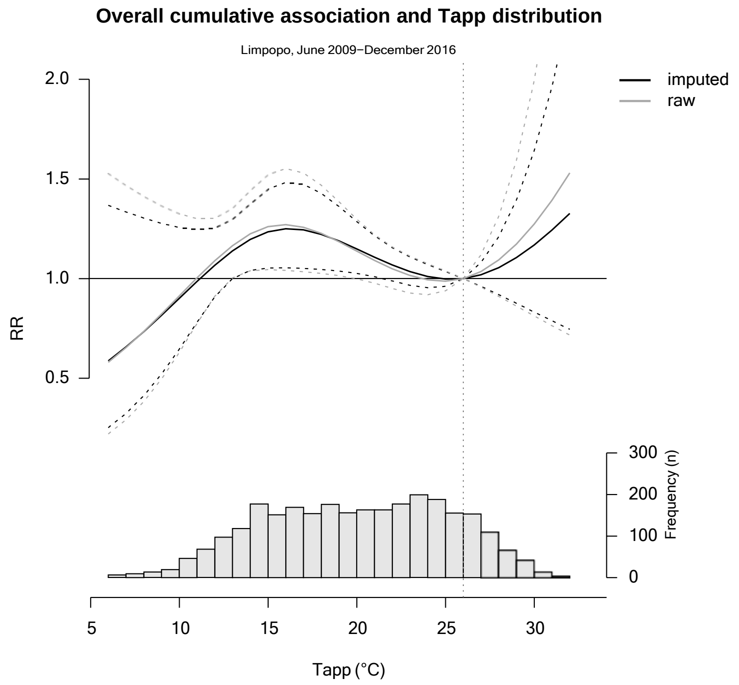

Figure S4: The relative risk (RR) of cardiovascular disease hospital admissions by apparent temperature (Tapp) cumulated over 21 days of lag, relative to 26°C in Limpopo, South Africa. The black and grey thick lines represent the RR using the imputed dataset (N=3,124) and raw dataset (N=2,371), respectively. The dotted lines represent the 95% confidence intervals and the grey vertical dotted line marks the optimum Tapp at 26°C. The histogram at the bottom shows the frequency of each Tapp occurring in Limpopo between 1.June 2009 and 31.December 2016.

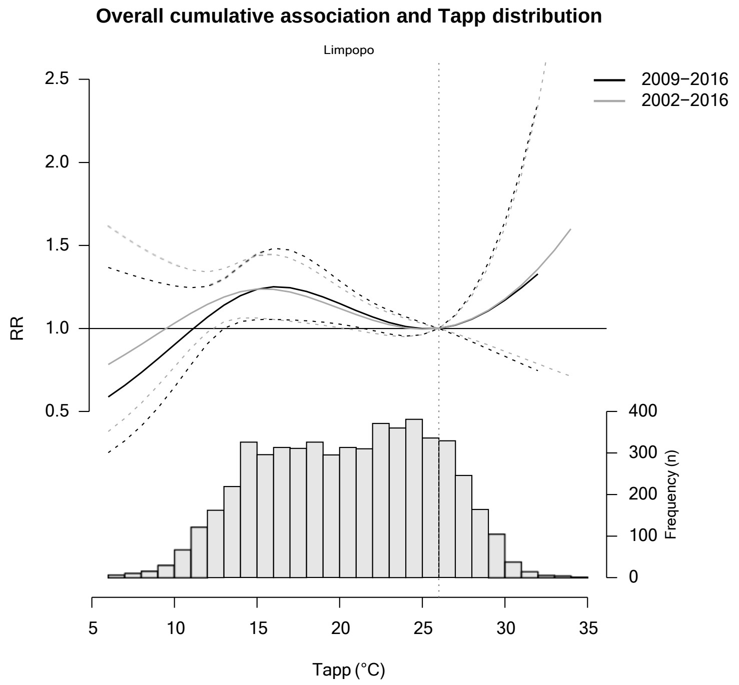

Figure S5: The relative risk (RR) of cardiovascular disease hospital admissions by apparent temperature (Tapp) cumulated over 21 days of lag, relative to 26°C in Limpopo, South Africa. The black and grey thick lines represent the RR using data from 1.June 2009 until 31.December 2016 (N=3,124) and non-imputed data from 1.January 2002 until 31.December 2016 (N=2,642), respectively. The dotted lines represent the 95% confidence intervals and the grey vertical dotted line marks the optimum Tapp at 26°C. The histogram at the bottom shows the frequency of each Tapp occurring in Limpopo between 1. January 2002 and 31. December 2016.
